# The Key Role of Vitamin D in Female Reproductive Health: A Narrative Review

**DOI:** 10.7759/cureus.65560

**Published:** 2024-07-28

**Authors:** Ramona E Dragomir, Oana D Toader, Daniela E Gheoca Mutu, Ruxandra V Stănculescu

**Affiliations:** 1 Doctoral School, Carol Davila University of Medicine and Pharmacy, Bucharest, ROU; 2 Obstetrics and Gynecology, Alessandrescu-Rusescu National Institute for Mother and Child Health, Polizu Hospital, Bucharest, ROU; 3 Obstetrics and Gynecology, Carol Davila University of Medicine and Pharmacy, Bucharest, ROU; 4 Anatomy and Plastic Surgery, Carol Davila University of Medicine and Pharmacy, Bucharest, ROU

**Keywords:** ergocalciferol, cholecalciferol, vitamin d status and pregnancy outcomes, vitamin d deficiency and hormonal balance, vitamin d and women's health, female fertility

## Abstract

Vitamin D, besides its crucial role in bone health and immune function, has received increased attention in recent years due to its possible impact on many processes related to female reproductive health. Recent research has tried to explain the role played by vitamin D in maintaining adequate hormonal status, fertility, and pregnancy outcomes. Our aim for this narrative literature review was to highlight and explain the mechanisms through which vitamin D status impacts female reproductive health. We believe this represents a very important subject of research, especially due to the increased incidence of infertility nowadays. Further studies are necessary on the association between vitamin D status and female reproductive health in order to fully understand its effects and to reach a consensus regarding vitamin D supplementation as a method to improve fertility status.

## Introduction and background

Female reproductive health includes the proper functioning of the reproductive system, menstrual cycle, hormonal balance, and the ability to conceive and sustain a pregnancy [1]. Various factors influence female reproductive health, including genetics, lifestyle, environmental exposures, and nutritional status. Recently, vitamin D status has been promoted as a significant factor regarding reproductive health due to its many biological roles.

To understand how vitamin D status affects female reproductive health, it is important to describe first its metabolism and principal effects on the human body. Vitamin D2 (ergocalciferol) and vitamin D3 (cholecalciferol) are the two most significant forms of vitamin D, each playing a crucial role in human health [2]. Sunlight exposure on the skin is the primary source of vitamin D, which can also be extracted from the diet, particularly in the forms of vitamin D2 and D3, as well as other less nutritious forms of vitamin D [3].

Vitamin D2 and D3 are converted into hormonally active forms through metabolism, first in the liver, then followed by the kidney. Due to its fat-soluble composition, vitamin D enters the bloodstream as a complex with a particular alpha1 globulin, known as the vitamin D transporter protein [4]. The first step in this process is the hydroxylation of vitamin D in the liver, producing 25-hydroxyvitamin D (25-(OH) D). The second hydroxylation of 25-OH vitamin D occurs in the kidney, resulting in the most important metabolite of vitamin D, 1,25-(OH)2 vitamin D, which is produced in the renal proximal tube by 1-alpha hydroxylase [5]. It's worth noting that the placenta and granulomatous tissue also play a significant role in synthesizing 1,25-(OH)2 vitamin D, serving as extrarenal locations for this process [5].

The main regulator of vitamin D metabolism is renal hydroxylation, a process influenced by blood calcium, phosphate, and parathyroid hormone (PTH) concentrations. Two significant factors, PTH and hypophosphatemia, work independently to enhance vitamin D 1,25-(OH)2 by increasing renal 1-alpha hydroxylase activity. Additionally, hypocalcemia boosts PTH secretion, increasing the kidneys' synthesis of 1,25-(OH)2 vitamin D [2-4]. The most accurate instrument for vitamin D status measurement is represented by 25 hydroxy-vitamin D.

Vitamin D optimal status is necessary for maintaining bone health, strengthening the immune system, and modulating several metabolic functions. According to Mayo Medical Laboratories, 25(OH)D serum values between 20-50 ng/mL represent optimal vitamin D status [6]. Despite its availability, vitamin D deficiency is a common health issue worldwide, affecting various populations, including women of reproductive age [1].

Vitamin D's effects on female reproductive health have been an important research topic recently. Adequate vitamin D levels have been associated with improved fertility, healthier pregnancies, and reduced risk of reproductive disorders such as polycystic ovary syndrome (PCOS) and endometriosis [1,7]. Studies have shown that vitamin D affects fertility through its role in the synthesis of sex hormones. Research from Yale found that only 7% of 67 infertile women had adequate vitamin D levels [7]. Simultaneously, research has demonstrated a strong correlation between the body's vitamin D levels and the success rate of in vitro fertilization. 

Besides the impact on fertility, vitamin D is also essential for pregnant women and infants. Recent research has indicated that optimal vitamin D status supports the development of the fetal skeleton and immune system and may reduce the risk of complications such as preeclampsia, gestational diabetes, and preterm birth [6,8,9]. For infants, optimal vitamin D levels are essential for healthy bone development and may reduce the risk of developing rickets and other health issues [1].

This narrative literature review aims to present the evidence linking vitamin D status to various aspects of female reproductive health. We conducted intensive research on original articles, reviews, and meta-analyses published in international databases such as PubMed, Web of Science, Cochrane, and Google Scholar. In order to appropriately identify the studies, we used keywords such as “vitamin D and health status”, “vitamin D and women's health”, “female reproductive health and vitamin D”, “vitamin D deficiency and hormonal balance”, “vitamin D status and pregnancy outcomes” and “vitamin D status and fertility". By synthesizing findings from observational studies, clinical trials, and meta-analyses, we sought to provide a comprehensive understanding of how vitamin D influences reproductive outcomes and to highlight potential implications for clinical practice. The conclusions of the included studies were summarized and analyzed using the narrative synthesis method in order to describe the results in a comprehensive way.

## Review

Vitamin D and general health status

Despite its classification as a vitamin, vitamin D functions more like a hormone, influencing various systems in the organism. Its importance extends beyond bone health, impacting immune function, cardiovascular health, mental health, and even chronic disease prevention.

The innate and adaptive immune systems, pancreatic β cells, and the circulatory and cerebral systems are among the targets of vitamin D [10]. Therefore, vitamin D can influence cell division and proliferation, immune response modiﬁcation, and hormonal production [11]. Taking into consideration its implication, vitamin D supplementation may be helpful in preventing and treating specific illnesses.

One of vitamin D's most well-known roles is maintaining bone health. It is known that conditions such as rickets in children and osteomalacia or osteoporosis in adults are direct consequences of vitamin D deficiency [12].

Due to its implication in regulating immune function, adequate vitamin D levels can help reduce the risk of various infections. Vitamin D optimal status may also play a role in preventing autoimmune diseases like multiple sclerosis and rheumatoid arthritis by lowering the level of inﬂammatory cytokines [12]. Studies have indicated that optimal vitamin D levels can positively impact the immune system by activating B cells, T cells, and antigen-presenting cells [13,14].

Regarding cardiovascular pathology, research indicated that an elevated risk of cardiovascular disease is linked to a poor level of vitamin D [15,16]. In a recent examination of the Framingham research, patients without a history of cardiovascular illness had their levels of 25-hydroxyvitamin D tested [2,16]. It was shown that patients with hypertension whose 25-hydroxyvitamin D level was less than 15 ng/ml had a 62% increased risk of cardiovascular events compared to those whose level was 15 ng/ml or higher [16-18].

According to research vitamin D receptors are also present in the brain and play an important role in brain development and function [19,20]. Vitamin D deficiency has been associated with an increased risk of depression, anxiety, and cognitive decline, therefore ensuring adequate vitamin D levels may support mental health and cognitive function, particularly in older adults [19,20].

These findings confirm the idea that vitamin D may have a key role in health status during different periods of life on many systems and support the need for further research regarding its actions.

The impact of vitamin D status on female reproductive health

Vitamin D Status and Menstrual Cycle Regulation

Nowadays, the majority of women of reproductive age have experienced at one point at least one episode of menstrual cycle dysregulation. This can be partially explained due to increased levels of everyday stress and external factors that can affect the hormonal axis. Studies have shown that vitamin D can regulate the menstrual cycle, and adequate vitamin D levels may help maintain normal ovulation and menstrual regularity. Vitamin D receptors (VDRs) found in human and animal granulosa and cumulus oophorus cells prove that Vitamin D is crucial in regulating the menstrual cycle [21,22].

Vitamin D's impact on hormonal regulation among women represents a hot topic of research and implies several physiological processes [23]. The primary hormone affected by vitamin D status is estrogen, and alteration of its normal levels leads to many dysregulations among women of reproductive age. Due to vitamin D binding to VDRs, the expression of genes involved in the synthesis and metabolism of estrogen is regulated by it. It was shown that vitamin D can increase the production of aromatase, which represents an essential enzyme involved in converting androgens to estrogen [23,24]. Vitamin D receptors are also present in the hypothalamus and pituitary gland, which suggests the possible influence of vitamin D on the release of gonadotropin-releasing hormone (GnRH) [25]. GnRH normal levels are essential for adequately releasing luteinizing hormone (LH) and follicle-stimulating hormone (FSH) from the pituitary gland, with a vital role in estrogen production and menstrual cycle regulation. Another important hormone influenced by vitamin D is represented by progesterone [23]. Several studies demonstrated that vitamin D increases the function of the corpus luteum, therefore ensuring adequate progesterone production, which is critical for menstrual cycle regulation [26].

Vitamin D also impacts insulin regulation, as shown by different studies [25,27]. It is widely known and accepted that increased insulin resistance is associated with menstrual cycle dysregulation. Vitamin D deficiency has been linked with increased insulin resistance, which can affect ovarian function and hormonal status [25,28].

It has also been associated with menstrual disorders, including polycystic ovary syndrome (PCOS), which is represented by irregular menstrual cycles and hyperandrogenism, mainly associated with insulin resistance [29]. A clinical trial by Salehpour et al. concluded that vitamin D supplementation in cases with deficiency can decrease insulin resistance in women with polycystic ovary syndrome [30]. Supplementing with vitamin D can also reduce the excessively high levels of anti-Mullerian hormone (AMH) in the blood and enhance the levels of anti-inflammatory soluble receptors for advanced glycation end-products in women with PCOS who have a deficiency in vitamin D [31]. Specifically, the administration of vitamin D and calcium, together with metformin treatment, in women diagnosed with PCOS may lead to positive outcomes in terms of monthly regularity and ovulation [31-33].

A 2018 study by Lagowska assessed vitamin D levels in the bloodstream and compared them with the menstrual cycle in young women [34]. It showed that decreased levels of 25(OH)D were linked to longer menstrual periods, namely oligomenorrhea or amenorrhea. Those with 25(OH)D levels below the recommended threshold of 30 ng/mL had nearly five times the likelihood of experiencing menstrual cycle problems compared to those with levels above 30 ng/mL [34].

Singh et al. found that women with regular menstrual cycles had notably elevated levels of vitamin D, whereas decreased levels of vitamin D were linked to a 13-fold increase in the likelihood of experiencing an irregular menstrual cycle [35].

Vitamin D Status and Fertility

As we know, in the past years, global fertility rates have been declining, representing a major health problem. It is known that lifestyle and environmental factors are directly implicated in this process. Additionally, sedentary lifestyles, poor diet, stress, and exposure to environmental toxins can adversely affect reproductive health. Conditions such as obesity, diabetes, and polycystic ovaries syndrome which have registered an increased incidence lately, have also a great impact on fertility [7].

The presence of vitamin D receptors in the ovaries, endometrium, and placenta suggests that vitamin D plays a role in reproductive processes [36,37]. As described above, the mechanism through which vitamin D status impacts fertility and hormonal status among women of reproductive age is complex [25]. Adequate vitamin D levels are associated with improved fertility outcomes, including higher conception rates and successful pregnancy development. Optimal vitamin D status is also linked to better outcomes in assisted reproductive technologies (ART), such as in vitro fertilization (IVF) [25]. Many studies have demonstrated a correlation between low vitamin D levels in the bloodstream and decreased natural fertility and the efficacy of IVF [29].

A particular subject of interest is vitamin D serum levels in follicular fluid [25]. A prospective study tried to establish if 25-hydroxyvitamin D serum values in the follicular fluid among women experiencing in-vitro fertilization are associated with the procedure's outcomes [38]. It was shown that optimal vitamin D status was correlated with an increased likelihood of achieving a pregnancy following in-vitro fertilization [38]. In contrast to these findings, another study investigating the values of 25-hydroxyvitamin D from follicular fluid found that insufficient vitamin D levels do not significantly impact the outcome of in-vitro fertilization [39]. The inconsistent findings may be related to the relatively small study group.

Zhou et al. studied the impact of vitamin D supplementation on pregnancy rates after in vitro fertilization in a meta-analysis from 2022. It involved five prior studies and concluded that achieving an optimal vitamin D status by using supplements improved the rate of chemical pregnancy, but regarding rates of clinical pregnancy, there wasn’t enough evidence [40]. 

The status of vitamin D before ovarian stimulation represents another important aspect of fertility research. A 2022 study conducted in Hong Kong analyzed this and concluded that after the first in-vitro fertilization cycle, the live birth rate was lower in cases of vitamin D deficiency than in optimal vitamin D status [41]. 

A meta-analysis conducted by Chenhao Xu et al. in 2024, consisting of 23 separate research, provides strong evidence indicating a possible association between blood vitamin D levels and the results of ART [42]. This research suggests that women who maintain adequate vitamin D levels are more likely to have successful live births, positive pregnancy tests, and clinical pregnancies after having ART treatments. On the other hand, those who have inadequate amounts of vitamin D have a lower likelihood of reaching these positive results [42].

Vitamin D Status and Pregnancy Outcomes

Despite the eﬀorts of several worldwide studies, there is still disagreement on the relationship between low vitamin D levels and the emergence of obstetric complications. Pregnant women often develop vitamin D insuﬃciency, shown in the blood levels of 25 hydroxyvitamin D in both the mother and the fetus. Especially when the need for vitamin D increases during pregnancy, a deﬁciency may harm the fetus's adequate development and the mother's health [1]. Recent research suggests that the eﬀects of vitamin D insuﬃciency during pregnancy may be considerably more signiﬁcant than previously believed.

According to studies, a balanced diet is insuﬃcient to meet a pregnant woman's needs for vitamin D, iron, and folic acid. Consequently, food consumption often does not surpass 2-2.2 μg of vitamin D per day, even though the daily need is at least 5 μg [43]. This is further supported by the observation that low levels of 25-hydroxyvitamin D (25-(OH)D), the most extensively used marker of vitamin D status regardless of source, are frequently found in pregnant women. Even in developed nations with less common nutritional rickets, vitamin D deficiency during pregnancy remains a signiﬁcant concern.

A moderate vitamin D deﬁcit is commonly deﬁned as a blood level of less than 20 ng/ml (50 nmol/L), while a severe deﬁciency is deﬁned as a level below 10 ng/ml. Mayo Medical Laboratories states that while 10-19 ng/mL indicates mild to severe insuﬃciency, the 25-hydroxyvitamin D2 and D3 value is ideal in the range of 20-50 ng/mL. A signiﬁcant insuﬃciency of vitamin D is indicated by levels below 10 ng/ml [6,10]. It is advised that pregnant women have an ideal circulating 25-(OH)D level of at least 40 ng/ml from the start of their pregnancy. [10] Research indicates that this level should oﬀer the highest level of protection against conditions such as the newborn's asthma or pregnancy-related complications such as preeclampsia [1]. It is crucial to remember that season, amount of sun exposure, and dietary habits may all aﬀect 25-hydroxyvitamin D levels.

**Figure 1:**
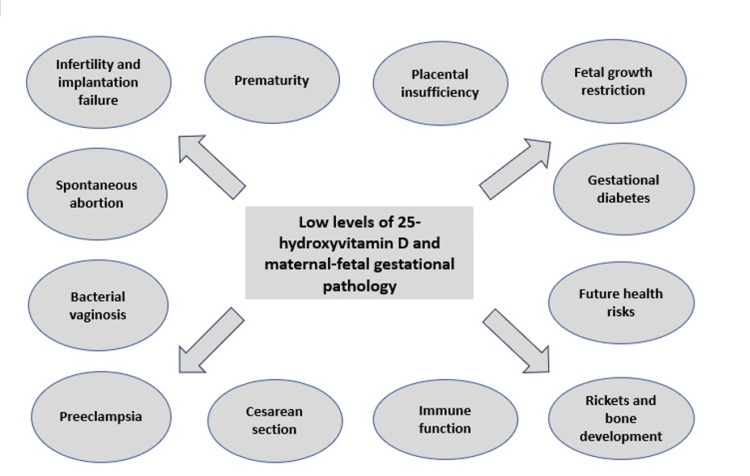
Vitamin D deficiency and pregnancy-related complications Image Credit: Dr. Dragomir E. Ramona

Even though vitamin D deficiency has been linked to osteoporosis and rickets, it is now widely acknowledged that vitamin D regulates many other biological processes. Certain tissues that can be broadly referred to as "barrier sites" have been found to express the VDR for the active form of vitamin D, 1,25-dihydroxy vitamin D (1,25(OH)2D), as well as the enzyme 1α-hydroxylase that synthesizes 1,25(OH)2D (CYP27B1) [37]. This suggests that particular responses to vitamin D may be a key feature of these tissues [44][45]. The placenta is the most common "barrier site". In the past, the placenta was among the earliest extrarenal tissues to produce 1,25(OH)2D, with fetal trophoblast and maternal decidua both presenting CYP27B1 activity [46,47]. International research has also shown the connection between vitamin D status and the placenta during pregnancy. Vitamin D modulates placental implantation, assisting in generating cytokines and the proper immunological response in the event of infection. On the other hand, the placenta manufactures vitamin D and reacts to its activity. Taking this into consideration, it is safe to say that vitamin D status can be associated with maternal-fetal complications during pregnancy [1,8]. The main pregnancy-related complications associated with vitamin D deficiency are presented in Figure 1 [1,8].

The benefits of vitamin D supplementation during pregnancy, either alone or combined with other micronutrients, were compared to a placebo or no intervention in a 2019 Cochrane study by Palacios [48]. Evidence from 22 trials totaling 3725 pregnancies indicates that pregnant women who only take vitamin D supplements may be at decreased risk of severe postpartum hemorrhage, low birth weight infants, gestational diabetes, and preeclampsia when compared to placebo or no intervention. According to the review's ﬁndings, vitamin D supplementation does not seem to have concrete evidence of lowering the risk of preterm delivery at less than 37 weeks gestation [5,11,48].

During pregnancy, there are signiﬁcant changes in the mother's calcium metabolism and vitamin D levels. Serum levels of 1,25 (OH)2D, which are larger in the case of pregnant women's circulation compared to the fetal circulation, might enhance the transit of vitamin D from mother to fetus, even though transplacental transport has not been examined in humans [44,45]. Pregnancy causes an increased 1,25(OH)2D production in the kidneys. The CYP27B1 enzyme also produces a signiﬁcant 1,25(OH)2D quantity in the decidua and placenta [37]. Furthermore, the transcription of this gene is repressed by speciﬁc methylation of placental CYP24A1 [47].

Studies have shown that vitamin D deﬁciency in gestational diabetes is likely largely inﬂuenced by increased CYP24A1 activity in the placenta. 25(OH)D is hydroxylated by CYP27B1 into the bioactive form 1,25(OH)2D, and CYP24A1 catabolizes both 25(OH)D and 1,25(OH)2D to inactive metabolites [49]. Furthermore, it has been proposed that vitamin D deﬁciency increases the likelihood of developing glucose intolerance. It was demonstrated that large dosages of vitamin D supplementation decrease insulin resistance in gestational diabetes-aﬀected pregnancies [50].

Vitamin D molecules' production, metabolism, and function during pregnancy are intricate processes. The human endometrial decidua produces 24.25(OH)2D while the placenta synthesizes 24.25(OH)2D [47]. The placenta's VDR content indicates that vitamin D may directly aﬀect certain tissues during pregnancy [51]. A plausible interpretation is that 1,25(OH)2D functions as a modulator of calcium transport in the placenta; nonetheless, the placenta's immunomodulatory function has also been suggested [51,52]. Furthermore, vitamin D may be essential for conception, implantation, and placenta formation due to the quick expression of VDR and CYP27B1 early in pregnancy [53]. In addition, vitamin D is critical in controlling perivascular support and neovascularization, suppressing placental neoangiogenesis and vascular development [54].

Many studies indicate that vitamin D insuﬃciency is linked to a higher risk of preeclampsia during pregnancy [55]. A substantial correlation has been shown in cross-sectional research between vitamin D deﬁciency and the likelihood of preeclampsia and its related consequences [56]. Vitamin D deficiency is described as one of the risk factors for the onset of preeclampsia [57]. The placental gene transcriptions are known to be altered by maternal physiological levels of 25(OH)D. This alteration includes decreased antiangiogenic factors, which may impact the risk of vascular complications during pregnancy. There is also evidence for an immunological cause and potential toxicity in preeclampsia linked to autoimmune diseases. The signiﬁcance of maintaining optimal vitamin D status throughout pregnancy to reduce the risk of preeclampsia is highlighted by these ﬁndings.

Vitamin D deficiency as a risk factor for intrauterine growth restriction and associated placental insufficiency was described in various studies. Even though intrauterine growth restriction has been mostly attributed to placental insufficiency, maternal malnutrition may also have an important contribution [58]. As we presented before, vitamin D has a key role in placental development; therefore, its impairment affects normal fetal growth and development. It was shown that the average vitamin D level of pregnant women whose newborns displayed intrauterine growth restriction was 33% lower than that of women whose babies displayed normal intrauterine development at delivery [59].

Another important topic is the fact that increased vitamin D serum levels have been linked in other studies to a decreased risk of both spontaneous and induced preterm birth brought on by a variety of medical conditions, including diabetes mellitus, hypertension, and prior preterm delivery. Research has also demonstrated that adequate vitamin D levels protect against miscarriages [60] and that women who had a normal pregnancy and delivery had considerably increased vitamin D status compared to those who had a spontaneous abortion [60,61]. Women who have repeated miscarriages and hypovitaminosis D are more likely to experience autoimmune diseases and cellular abnormalities than women who experience repeated miscarriages and normal vitamin D levels [60].

It was described that pregnant women who consume less than 400 IU of vitamin D daily are at a heightened risk of miscarrying [62]. Furthermore, a study compared women with low 25(OH)D levels to those with a level of 26.4 ng/ml, and it was revealed that in cases with adequate vitamin D status, there was a four-fold increased risk of successful reproduction [63]. According to a study by Hollis et al., women who received 4,000 IU of vitamin D per day as dietary supplements had a 50% lower risk of preterm delivery and a 25% lower risk of maternal infection than those who received 400-2000 IU per day [64].

Due to vitamin D's role in immune functions, bacterial vaginosis has been linked to a higher risk in pregnancies associated with vitamin D deficiency [65]. This condition can cause discomfort to pregnant women and affect the viability of a pregnancy [66]. In addition, miscarriages, early membrane rupture and preterm delivery, chorioamnionitis, and postpartum endometritis have been linked to bacterial vaginosis [65,66]. Eﬀective, adequate antibacterial, and anti-inﬂammatory responses are necessary for a normal pregnancy [65]. Studies have shown that adequate vitamin D levels enhance the body's ability to ﬁght oﬀ infections in both the placenta and the genital tract [65]. In one research, for instance, 440 pregnant women were shown to have a three-fold increased risk of bacterial vaginosis if their blood 25(OH)D levels were below 30 ng/mL [66].

It is thought that low levels of vitamin D in maternal serum also raise the possibility of cesarean delivery [67]. According to one research, women with a 25(OH)D level below 15.2 ng/ml had a roughly four-fold higher incidence of cesarean sections than those with a level over 15.2 ng/ml [68]. Methodological variability across studies and the wide range of reasons for elective and emergency cesarean sections severely limit a more appropriate assessment of the influence of vitamin D on the completion of cesarean procedures. Therefore, currently, there is a lack of sufficient data to prove the association between a pregnant woman's risk of cesarean birth and a vitamin D deficiency.

Taking into consideration the link between vitamin D deficiency and the multitude of pregnancy-related complications, further research and randomized clinical trials are necessary to fully understand the underlying mechanisms.

## Conclusions

Vitamin D status greatly impacts female reproductive health by influencing menstrual cycle, fertility, and pregnancy outcomes. To maintain an optimal health status among fertile women, adequate vitamin D levels must be ensured through a combination of sunlight exposure, diet, and supplementation.

Given the high incidence of vitamin D deficiency, especially in pregnant women, monitoring and managing vitamin D status should be a “gold standard” for any medical practitioner. Further research is still needed to reach a consensus on the impact of vitamin D status on female reproductive health and to completely understand the mechanism that influences fertility and pregnancy-related complications.
